# Post-Covid Syndrome: A standardized assessment on subjective psychiatric and neuropsychological symptoms

**DOI:** 10.1192/j.eurpsy.2022.1355

**Published:** 2022-09-01

**Authors:** D. Roesch Ely, B. Kramer, A. Jaehn, U. Merle, M. Weisbrod

**Affiliations:** 1 University of Heidelberg, General Psychiatry, Heidelberg, Germany; 2 University of Heidelberg, Internal Medicine - Gastroenterology, Heidelberg, Germany

**Keywords:** Long COVID, psychiatry, Neuropsychology

## Abstract

**Introduction:**

Long-Covid or Post-COVID-19 syndrome develops during or after an infection with COVID-19 and continues for more than 12 weeks. The signs and symptoms are not explained by an alternative diagnosis. Neuropsychiatric symptoms are usually manifested as cognitive impairment (brain fog, loss of concentration or memory issues, etc.), headache, sleep disturbance, peripheral neuropathy symptoms (pins and needles and numbness), dizziness, anosmia, symptoms of depression, anxiety and fatigue. Patients complain of reduced quality of life and impairment on daily functioning. Although the burden of disease is high there is until now very few data available, the etiopathology is still unknown and treatment strategies are not established.

**Objectives:**

The objective of this study is to gather standardized data of patients with long-covid syndrome who suffer from neuropsychiatric symptoms in order to better understand the complexity of this syndrome.

**Methods:**

Patients were referred from the long-covid outpatient unit of the internal medicine department to our specialized outpatient unit, so that the previous infection was confirmed. A standardized psychiatric interview and a thorough neuropsychological assessment was conducted.

**Results:**

We will present preliminary data on psychiatric symptoms, neuropsychology and quality of life with patients with long-covid syndrome.

**Conclusions:**

Potential treatment strategies to improve psychiatric and neurocognitive symptoms as well as improvement of quality of life will be discussed.

**Disclosure:**

Daniela Roesch Ely and Matthias Weisbrod have a contract with Schuhfried GmbH (development of neurocognitive batteries and training programs)

